# The Role of Artificial Intelligence in Healthcare: Enhancing Coronary Computed Tomography Angiography for Coronary Artery Disease Management

**DOI:** 10.7759/cureus.61523

**Published:** 2024-06-02

**Authors:** Dhammadam Thribhuvan Reddy, Inayat Grewal, Luisa Fernanda García Pinzon, Bhargavi Latchireddy, Simran Goraya, Badriya Ali Alansari, Aishwarya Gadwal

**Affiliations:** 1 Department of Medicine, Columbus Central University, Belize City, BLZ; 2 Department of Medicine, Government Medical College and Hospital, Chandigarh, IND; 3 Department of Medicine, Fundación Universitaria de Ciencias de la Salud, Bogotá, COL; 4 Department of Gastroenterology, Craigavon Area Hospital, Craigavon, GBR; 5 Department of Medicine, Kharkiv National Medical University, Kharkiv, UKR; 6 Department of Medicine, Gulf Medical University, Sharjah, ARE; 7 Department of Radiodiagnosis, St. John’s Medical College and Hospital, Bengaluru, IND

**Keywords:** ai in cardiology, ai in ccta, ai in diagnosing diseases, deep learning, machine learning, medical imaging analysis, diagnostic imaging, coronary computed tomography angiography (ccta), coronary artery disease (cad), artificial intelligence (ai)

## Abstract

This review aims to explore the potential of artificial intelligence (AI) in coronary CT angiography (CCTA), a key tool for diagnosing coronary artery disease (CAD). Because CAD is still a major cause of death worldwide, effective and accurate diagnostic methods are required to identify and manage the condition. CCTA certainly is a noninvasive alternative for diagnosing CAD, but it requires a large amount of data as input. We intend to discuss the idea of incorporating AI into CCTA, which enhances its diagnostic accuracy and operational efficiency. Using such AI technologies as machine learning (ML) and deep learning (DL) tools, CCTA images are automated to perfection and the analysis is significantly refined. It enables the characterization of a plaque, assesses the severity of the stenosis, and makes more accurate risk stratifications than traditional methods, with pinpoint accuracy.

Automating routine tasks through AI-driven CCTA will reduce the radiologists' workload considerably, which is a standard benefit of such technologies. More importantly, it would enable radiologists to allocate more time and expertise to complex cases, thereby improving overall patient care. However, the field of AI in CCTA is not without its challenges, which include data protection, algorithm transparency, as well as criteria for standardization encoding. Despite such obstacles, it appears that the integration of AI technology into CCTA in the future holds great promise for keeping CAD itself in check, thereby aiding the fight against this disease and begetting better clinical outcomes and more optimized modes of healthcare. Future research on AI algorithms for CCTA, making ethical use of AI, and thereby overcoming the technical and clinical barriers to widespread adoption of this new tool, will hopefully pave the way for profound AI-driven transformations in healthcare.

## Introduction and background

While digital subtraction angiography is the gold standard test for diagnosing coronary artery disease (CAD), it has its limitations: its invasiveness, its high cost, and its inability to detect the constitution and identity of the plaques [1]. Hence, coronary CT angiography (CCTA) has emerged as an alternative imaging modality for diagnosing CAD. CCTA has several benefits, such as being noninvasive, convenient, quick, and relatively affordable, making it very suitable for clinical CAD screening [2]. Recent years have witnessed an evident rise in CCTA examinations, leading to an increase in cardiac imaging data. This surge has drawn attention to the challenges associated with it, including a shortage of radiologists, which results in a decrease in image quality, a rise in misdiagnosed cases, and delays in reports. With CCTA poised to play an increasingly substantial role in ruling out CAD, there is increasing pressure on radiologists to manage the large workload efficiently.

In the medical field, cardiac imaging and other artificial technology have proven to be revolutionary. Many artificial intelligence (AI) algorithms and methods have been developed, further enhancing the automatic imaging technology that can be employed for a more accurate diagnosis as well as in devising personalized treatment plans. These algorithms have significantly improved the clinical application of cardiological imaging by aiding in image recognition, cutting, etc. [3]. In this review, we discuss how AI helps with earlier diagnosis of CAD through CCTA. We commence by touching on certain basic ideas about AI, its involvement in various fields, as well as its hardware side. We also engage in a thorough analysis of the current problems associated with the technology and the future of the integration of AI into CCTA, as well as AI's broader role in healthcare solutions going forward.

## Review

AI in medicine

AI technology is widely expected to revolutionize fields such as healthcare, business, IT, education, finance, and the entertainment industry, and the term refers to computer systems that copy or look like humans. AI is used extensively in medicine; it is employed to analyze medical data and offer critical insights into various aspects of patient care, including clinical judgment, decision-making, and patient outcomes. It may also be used in education and continued exploration. Its major goals include clinical judgment, analysis and interpretation of images, higher-level decision-making, and formulation of treatment plans. Several hospitals worldwide use AI to manage large datasets of their EHR systems, another beneficial source for the healthcare provider, which includes the patient’s medical record of previous admissions, prior diseases, allergies, current dosage, blood records, and prior imaging findings. In addition, it has transformed medical management, medical treatment, and drug production [4,5].

AI in Medical Diagnosis and Imaging

AI is employed in diagnosing illnesses via radiological imaging, pathology, endoscopic procedures, and biochemical laboratory tests. Radiology is essential in healthcare, providing a vital basis for identifying various diseases. The need for radiological assessments is increasing annually. Nevertheless, training proficient healthcare workers in this area is time-consuming, and the number of doctors specializing in radiation medicine is expanding sluggishly. Hence, the disparity between the availability and demand of medical professionals in radiology is rising, leading to elevated job-related stress and misdiagnosis rates. In light of these obstacles, it is crucial to investigate alternative approaches, like utilizing AI, to tackle this urgent issue [6]. A branch of AI known as "machine learning" (ML) uses algorithms trained on data sets to build models that allow machines to do tasks like identifying images and data analysis, tasks that are otherwise only achievable by humans.

**Figure 1:**
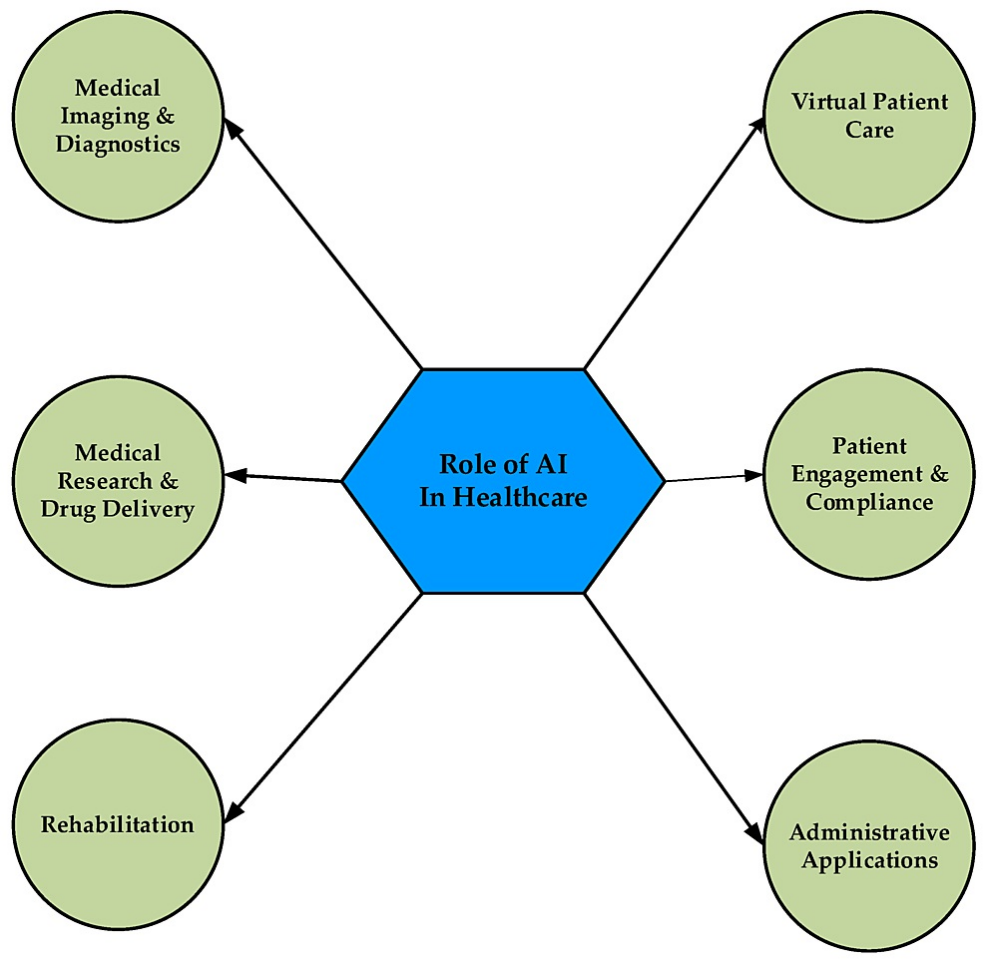
Diagram depicting the role of artificial intelligence in healthcare Reproduced under the terms and conditions of the Creative Commons Attribution (CC BY) license from reference [9]. Copyright © 2023 by the authors. Licensee MDPI, Basel, Switzerland.

AI encompasses many techniques, with ML constituting a particularly significant approach in medicine. The utilization of ML in medical applications has been greatly facilitated by three key technical advancements: (a) the emergence of 'big data', which involves the creation and analysis of extensive databases; (b) the remarkable enhancement in computing power of processors, and (c) the development of innovative deep learning algorithms [7]. Advanced ML and deep learning methods are currently being implemented in CT, MRI, PET-CT, and other imaging modalities, demonstrating remarkable efficacy in various applications such as image segmentation, classification, reconstruction, and registration [8]. Medical radiation practitioners are at the forefront of the ML revolution. The traditional medical model, which cannot provide prompt and correct findings, particularly for complex diagnoses, can be replaced by AI, which can produce results rapidly. Furthermore, because AI can resolve problems at a rapid pace, doctors may be able to develop more considerate and practical treatment regimens depending on the circumstances of the patient (Figure 1).

AI is also employed in wearables such as timepieces, saturation probes, and blood pressure devices. The utilization of wearables for health monitoring has witnessed a surge in recent years, particularly in the wake of the coronavirus disease 2019 (COVID-19) pandemic. Miyashita and Brady provided an example where discharged patients were equipped with a Wi-Fi-enabled armband that remotely tracks essential health indicators, including respiratory rate, oxygen levels, pulse, blood pressure, and body temperature [10]. This initiative was implemented across a cluster of hospitals catering to a population of 500,000 people in southeast England. This initiative to apply AI programs that analyze patient data in real time led to a significant decrease in hospital readmission rates and emergency room visits. Moreover, costly home visits were reduced by 22%. In the long run, adherence to the treatment plan witnessed a remarkable increase to 96%, surpassing the industry average of 50% [11]. In the future, AI will play a crucial role in the field of medicine. Therefore, it is imperative to educate the new generation of medical trainees about the principles and practicality of AI and how to collaborate with machines to enhance productivity effectively.

Coronary artery disease

Epidemiology

CAD is the most significant single cause of mortality and loss of disability-adjusted life years (DALYs) worldwide, accounting for roughly seven million deaths and 129 million DALYs annually [12]. CAD is a high-cost disease and is estimated to account for one-third of a projected $47 trillion in economic losses due to non-communicable diseases over the next 20 years. According to the American Heart Association (AHA), heart disease costs the United States about $219 billion each year. In 2020, CAD accounted for approximately 41.2% of cardiovascular diseases, making it the leading cause of death (382,820) in the United States [13]. Comparing the rate of deaths from coronary heart disease between 2010 and 2020, a 19.2% decrease in annual deaths was observed. However, globally, noteworthy increments in the rate of cardiac disease have been reported in Latin America and the Middle East. The high rate of CAD in Latin America is explained by an inactive way of life, smoking, and overweight. Another study has analyzed the data provided by the WHO on coronary heart disease; in four of the countries evaluated (United Kingdom, Ukraine, Kazakhstan, and Brazil), a higher mortality rate in adults over 80 years of age was observed in 2017 [14].

Pathophysiology

**Figure 2:**
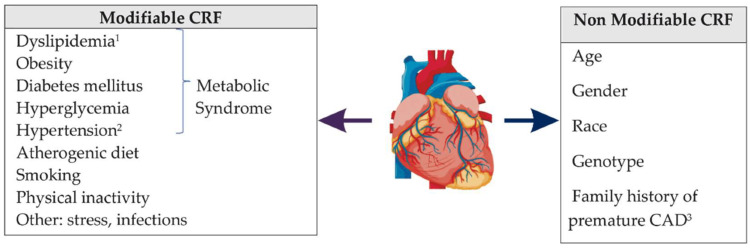
Classification of cardiovascular risk factors (CRF) ^1^Dyslipidemia is a condition characterized by abnormal levels of lipids in the blood, including total cholesterol more than 200 mg/dL, LDL-C greater than 130 mg/dL, HDL-C less than 40 mg/dL, and triglycerides greater than 150 mg/dL [17].^2^Hypertension is characterized by blood pressure (BP) equal to or more than 140/90 mmHg according to the European standards [18]. In contrast, the American criteria utilize a lower threshold of BP equal to or greater than 130/80 mmHg [19].^3^There is a history of premature coronary artery disease (CAD) in the user's family, namely in a male first-degree relative under the age of 55 or a female first-degree relative under the age of 65. This information is supported by [20] LDL-C: low-density lipoprotein-cholesterol; HDL-C: high-density lipoprotein-cholesterol Reproduced under the terms and conditions of the Creative Commons Attribution (CC BY) license [16]. Copyright © 2022 by the authors. Licensee MDPI, Basel, Switzerland

The development of CAD is associated with multiple risk factors (Figure 2), both non-modifiable and modifiable (Figure 2), and it is evident that smokers have a mortality risk greater than 70%, mainly resulting from obstruction of blood flow, which leads to a mismatch between myocardial oxygen demand and supply. This occurs due to atherosclerotic plaque formation; when there is a vascular insult, the intima layer breaks, allowing monocyte migration into the subendothelial space to become macrophages [15].

These macrophages take up oxidized LDL particles and foamy cells are formed. These foamy‐texture cells replicate and form lesions (fatty streak), generating the accumulation of extracellular matrix and leading to the progression of the lesion to fibrous plaque, which infringes on the lumen of the coronary vessel, and small blood vessels are formed. The final lesion formed includes a fibrous cap with an overlying lipid‐rich core containing necrotic material that may be highly thrombogenic. This plaque may grow in size or become stable if no further insult occurs to the endothelium. When the plaque suffers a rupture, it leads to the exposure of tissue factor, which culminates in thrombosis and requires stenosis of more than 90% of the blood lumen to become hemodynamically significant and it could result in acute coronary syndrome (ACS) [angina, ST-elevation myocardial infarction (STEMI), or Non-ST-elevation myocardial infarction (NSTEMI)] [16].

Clinical Features

It is crucial to investigate the most common symptoms and their relationship with physical activity, family history, and lifestyle habits. A complete physical examination that includes inspection, palpation, and auscultation should be conducted. The most common symptoms include retrosternal chest pain, syncope, palpitations, tachypnea, lower extremity edema, orthopnea, and decreased exercise capacity. A physical examination can show any acute distress, jugular venous distension, and peripheral edema in advanced disease [15].

Diagnosis

The diagnosis of coronary heart disease begins with assessing the clinical presentation; however, an essential diagnostic aid is the EKG, which provides information about the anatomy and physiological functions of the heart. The changes evident in acute coronary disease in this diagnostic tool are changes in the ST segment and the T wave; advanced acute coronary disease can result in arrhythmia. In chronic settings, EKG can show aspects like axis deviation, bundle branch blocks, and ventricular hypertrophy. If the EKG does not show ST-segment elevation, troponins should be taken, which can generate two extreme values. The first group may correspond to an exaggerated elevation of these (>5 times the average value), indicating a positive diagnosis. The second group corresponds to deficient levels of troponins; therefore, ACS should be ruled out if the pain has been present for more than three hours. If the patient's troponin values ​​do not correspond to any of the previous groups, a new ultrasensitive troponin measurement must be performed in the following hour. ACS is diagnosed based on a difference of >20% to the initial troponin levels [21,22].

According to the AHA, the use of emergency echocardiography is only recommended in patients with suspected CAD who present with cardiogenic shock or suspected mechanical complications. Likewise, the use of this tool is indicated in chronic settings to identify the response to the therapy and the possible complications [23]. The use of CT angiography of coronary arteries has an IIa recommendation for patients in whom ACS is suspected without EKG changes (absence of ST elevation) or recurrence of pain [24]. Despite being an invasive technique, cardiac catheterization is the gold standard tool for ischemic coronary disease. Patients with an intermediate probability of CAD tend to be the best candidates to undergo this procedure. In the acute presentation, all patients with STEMI and selected NSTEMI are candidates for emergency catheterization.

Treatment

According to the AHA, the condition is defined as the presence of substernal pain that radiates to the jaw, which is caused by physical activity or stress and relieved with rest or nitrates. The management of this presentation is based on lifestyle modifications including smoking cessation, regular exercise, weight loss, reasonable control of diabetes and hypertension, and a healthy diet [25]. ACS is defined by the AHA as the elevation or decrease of troponins above the 90th percentile accompanied by ischemic symptoms, and electrocardiographic and echocardiographic changes (visualization of the thrombus). The management for STEMI presentation involves an emergency percutaneous coronary intervention (PCI) if the facility is equipped for this procedure or if it is available within a distance of two hours; if the facility with PCI is more than two hours away, intravenous thrombolytic therapy is indicated provided there are no contraindications to it. The usefulness of PCI is based on opening a blocked artery and reducing the amount of cardiac damage. Carrying out this procedure requires trained personnel and is based on inserting and inflating a tiny balloon where the artery is clogged to enlarge it and to decrease the chance of artery contraction over again; a stent is placed permanently to keep the artery open [26].

The basic concept of AI in clinical medicine

AI and Its Growing Application in Various Domains

The field of AI involves constructing machines capable of performing operations that might typically require the abilities of the human mind [27]. Models and algorithms utilized in AI are designed to resemble the thoughts and movements of humans. It has advanced considerably in several industries, including healthcare, banking, transportation, and education [28]. AI is utilized in algorithmic trading, self-driving automobiles, risk assessment, fraud detection, infection prediction, drug development, and personalized learning [29]. Its growing importance results from its capacity to absorb and utilize extensive amounts of facts, perform tricky computations, gain understanding, and outperform people at duties in terms of accuracy and efficiency. AI's role is expected to grow even further as technology develops, creating new possibilities for improvement and innovation.

Growing Use of AI in Clinical Practice

A semi-supervised system was created by Ashdown et al. to categorize malaria parasite tiers in step with morphological variability [30]. The development of Plasmodium falciparum and the consequences of antimalarial medicines have been defined by using this version. The version generated 384-dimensional embeddings using unlabelled facts and human labeling. According to the researchers, over 650,000 versions had higher rankings with illness annotation, and 94% of variants related to altered splicing had been accurately classified. The deep neural network (DNN) visualized on-cycle medication effects and diagnosed outliers at some point of asexual improvement [31]. The researchers' work from 2021 investigates the use of on-chip synthesis and generative AI for de novo drug creation [32]. Through a design-make-test-analysis cycle, the researchers learned much about and used on-chip chemical synthesis microfluidics technology to make liver X receptor (LXR) agonists for the liver. The generated LXRs were powerful, indicating a novel remedy technique that warrants additional research. After being taught with 656,070 molecules, 40 well-known LXRα agonists were used to fine-tune the generative deep learning model. Using high-performance liquid chromatography coupled with tandem mass spectrometry (HPLC-MS), the very last drug structure was tested and examined [33,34]. Evidently, within clinics and hospitals, a revolution is subtly taking shape.

AI is rapidly becoming a key component of contemporary medical practice, and it is far from just a sci-fi dream [35]. AI programs were regarded as something from a science fiction film only a few years ago. These days, those technologies directly affect how we diagnose and treat ailments [36]. This evaluation explores the transformational potential of AI by delving into its essential ideas. It is now possible to imagine a future in which AI algorithms possess superhuman precision in analyzing scientific scans and figuring out minute irregularities that may go unnoticed by people [28]. This is not a pipe dream thanks to AI's prowess in image identity. AI can also analyze large volumes of clinical facts to locate patterns and developments that would enable early damage prediction and more focused treatment regimens. There are many possible benefits, such as better patient outcomes and a more effective healthcare system. However, integrating AI into medicine faces several challenges. Sensitive patient data must be blanketed with robust protections, making data privacy and protection top priorities. Furthermore, developing trust in the correctness of AI models relies on assuring their explainability or the capacity to realize how they get their findings [35]. Notwithstanding these barriers, there is no denying AI's growing use in medical practice. This review examines the several ways AI is changing healthcare, presenting particular benefits and emphasizing viable merits. In short, AI can usher in a new age of medical care marked by increased accuracy, performance, and, ultimately, superior outcomes overall.

AI Subfields With Applications in Medicine

AI's transformational potential in medical care is linked to its various applications. In this review work, we study three essential subfields and how they are utilized in the medical environment

Machine learning (ML): ML structures can "research" vast volumes of medical data, viewing links and tendencies that humans could forget, enabling them to forecast, recommend, and suggest various patterns. For instance, an ML model created based on patient records would possibly forecast the probability of contracting a certain illness, thereby allowing early intervention [37].

Deep learning (DL): DL refers to a branch of ML that handles complicated facts, including clinical pictures, by using an artificial neural network (ANN) that approximates the structure of the human mind. For example, DL algorithms can analyze medical pictures and accurately figure out anomalies, including tumors or fractures, thus facilitating faster analysis. Machines can now recognize and interpret human language according to natural language processing (NLP). For example, NLP may evaluate clinical notes and medical statistics, obtain essential information, and streamline data analysis for studies or individualized remedy regimens (Table 1) [38,39].

**Table 1:** AI subfields in clinical medicine ML: machine learning; DL: deep learning; ANN: artificial neural network; NLP: natural language processing; RL: reinforcement learning

Subfield	Function	Clinical use	Source
ML	Use unique characteristics to find patterns that may be utilized to examine a given circumstance	Risk assessment and disease prognosis	[40]
DL	A branch of ML that makes use of multilayer neural networks	Drug discovery and analysis of imaging in medicine	[41]
NLP	Focuses on natural language interactions between computers and people	Evaluation of patient requirements and clinical decision assistance	[42]
RL	A branch of ML in which a system learns to make decisions by operating in a manner that optimizes value in its surroundings	Individualized care regimens and automated operations	[43]

Since large amounts of patient information, including imaging scans, lab tests, and health records, among others, are processed by the trained systems, the structures may be used to detect trends and potential diagnoses. As a result, it may be helpful for doctors in predicting dangers, early diagnosis of diseases, and personalized treatment alternatives. Another example involves trained devices that can accurately analyze a chest X-ray to aid radiologists in detecting lung diseases like pneumonia [44,45]. DL has proved to be revolutionary in the field of clinical imaging analysis. DL algorithms can be instructed by using extensive patient pictures, including X-rays, CT scans, and MRIs, to recognize abnormalities with high precision. This may reduce false positives, speed up diagnosis, and improve the precision of cancer detection.

AI can also handle automatic picture-processing tasks, freeing up radiologists, thus enabling them to tackle tough cases and enhance their responses to patients. The amount of effort and money that goes into the traditional drug discovery procedure is incredible. Incorporating AI opens up new pathways to accelerate and fine-tune this technique. ML algorithms may examine vast amounts of biological and chemical component data to find potential drug applicants. AI-powered tools may also anticipate the efficiency and potential adverse consequences of compounds to aid in the drug development process [46]. AI can enhance medical trial design and optimization. Trials can be performed more efficaciously and informatively using ML algorithms to find possible volunteers who are more likely to react to a positive therapy. AI can likewise perform real-time analysis of clinical trial records, which hastens the technique of identifying feasible benefits or safety-related issues (Table 2) [47,48].

**Table 2:** Features and possible benefits of using AI AI: artificial intelligence; ML: machine learning; DL: deep learning; MRI: magnetic resonance imaging; CT: computed tomography

AI utilization	Function	Prospective merits	Ref.
Devices for diagnostic aid	ML algorithms examine patient data to find trends and possible diagnoses	Better risk assessment, early illness identification, and individualized treatment planning	[49]
Evaluation of medical imaging	To find abnormalities, DL algorithms examine medical pictures such as CT scans, X-rays, and MRI	Enhanced cancer detection, quicker and more accurate diagnostics, and fewer false positives	[50]
Drug production and research	Large-scale datasets are analyzed by ML algorithms to find potential candidates for medication and forecast side effects and effectiveness	Quicker and more efficient drug development, as well as safer and stronger medications	[51]
Design and optimization of clinical trials	Real-time trial data analysis and participant identification are performed by ML techniques	Trials are conducted more effectively and informatively, with possible advantages or safety issues being identified more quickly	[52]
Individualized medical care	AI examines individual patient data to forecast therapy outcomes and suggests tailored treatment regimens	Customized care plans that enhance patient outcomes and treatment effectiveness	[53]
Robotic surgery	Robotic technologies driven by AI let surgeons do less invasive surgeries	Quicker patient recovery times, fewer problems, and increased surgical accuracy	[53]

Current status of AI use in coronary CT scanning

Artificial intelligence has shown great potential in improving diagnosis and prognosis in patients with cardiovascular disease receiving coronary CT scans. CT examination analysis may now be completed more quickly and reliably thanks to recent developments in DL, which have made it possible to extract features from big datasets and learn from them automatically. Automated coronary plaque segmentation, total plaque volume measurement, coronary artery calcium assessment, measurement of epicardial fat, and cardiac event prediction are among the AI applications used in cardiovascular CT. AI has also been used to estimate coronary stenosis and plaque, evaluate calcium score and heart volumes, characterize myocardial tissue in cardiac CT scans, and examine heart volumes to evaluate the cardiac chambers' size and capacity [54].

As we move forward, noninvasive imaging is gaining prominence as a vital component of diagnosing ailments while invasive method remains the go-to method for pinpointing and addressing CAD; techniques like cardiovascular CT, notably CT coronary angiography, are poised to play a more significant role in ruling out CAD, and this shift will likely lead to a more significant workload for radiologists in the future. It is then that AI, which involves intelligent computer programs that learn and understand patterns like ML and DL along with techniques that extract numbers and details from medical images like CT scans called radiomics, could help with reducing the workload [54].

**Figure 3:**
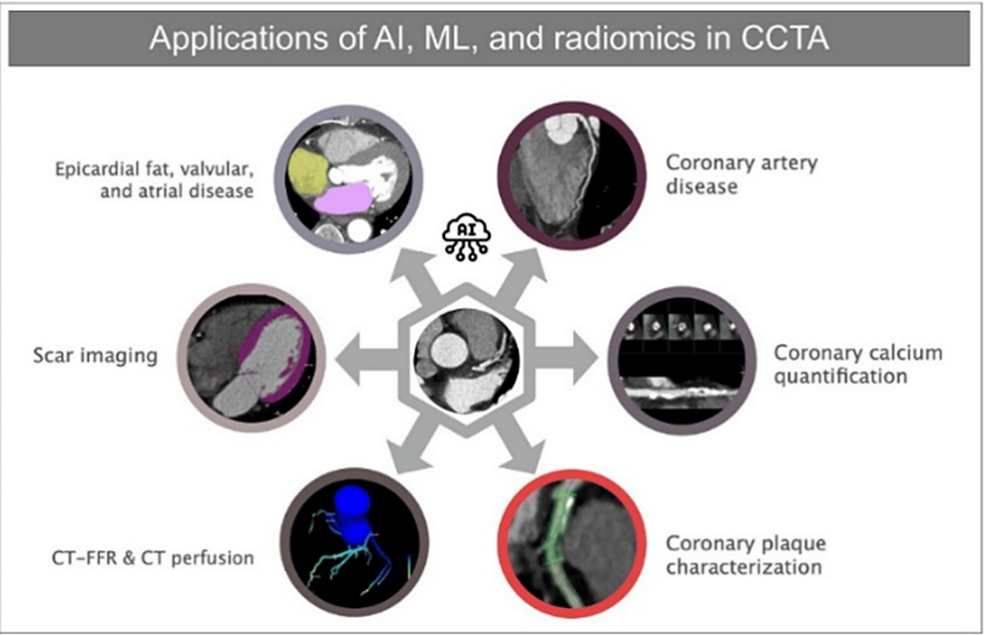
The use of artificial intelligence, machine learning, and radiomics in coronary CT angiography for various purposes AI: artificial intelligence; ML: machine learning; CCTA: coronary computed tomography angiography; FFR: fractional flow reserve Reproduced under the terms and conditions of the Creative Commons Attribution License (CC BY) [55]. Copyright © 2023 Baeßler, Götz, Antoniades, Heidenreich, Leiner and Beer

Using AI, we can detect and describe abnormalities in the coronary artery CT images, such as calcified or non-calcified plaques, coronary artery constriction, or other noteworthy findings. AI can also predict the likelihood of future heart issues by studying the unusual things observed in CT scans. This means that doctors can use AI to figure out which patients might have heart problems in the future (Figure 3) [55].

As people get older, their chances of having coronary artery calcification (CAC) increase significantly, especially in men. This is important because CAC is a sign of heart disease and can predict future heart problems. However, even though CAC tests are helpful, they can be complicated and time-consuming, and they are not often performed in busy hospitals. This is where AI-based systems come in. These systems can accurately measure CAC without needing any extra work from doctors or causing any discomfort to the patient. This means that patients can still get the care they need even in busy hospitals. By interpreting images, AI can also help identify different types of skin cancer, detect cerebral aneurysms, guide treatment choices for cerebellar infarction, and predict fragility fractures and osteoporosis. These advancements suggest that integrating AI into CAC and risk assessment will likely become essential [56].

AI-Assisted Diagnosis of CCTA Findings

CCTA is commonly used to diagnose CAD. AI tool's functions go beyond CAD recognition itself; it is also used to follow and evaluate coronary stenosis, plaque characterization, plaque buildup, the extent to which the coronary arteries are narrowed, and the assessment of myocardial ischemia, all of which could lead to CAD development. CCTA can be utilized to strategize suspected CAD patients for risk stratification [57]. Based on CCTA findings, patients can be classified according to their risk of CAD development in the future, which helps physicians put patients on early therapy and medications. CCTA-based prognosis has been reported to reduce non-fatal as well as fatal myocardial infarctions. Although CCTA is very capable of detecting CAD, it also has certain challenges, such as its time-consuming nature, its requirement for the utilization of advanced postprocedural techniques, and the occasional exaggeration of the degree of stenosis. Therefore, AI is a helpful tool to reduce the reporting time and detect coronary lesions that can cause ischemia. Various studies have applied AI to detect the degree of stenosis.

Kang et al. introduced an AI system with a two-step algorithm using vector machines, which proved useful in detecting CAD stenosis. The study involved 42 patients who underwent dual-source CT imaging, and their algorithm showed the capability to detect stenosis within one second with a specificity of 95%, a sensitivity of 93%, and an accuracy of 94%. Zreik et al. employed a 3D convolutional neural network (CNN) capable of characterizing plaque and assessing stenosis [58]. They developed two models, the first aimed to distinguish patients with and without obstructive CAD, achieving a patient, per segment, and vessel accuracy of 0.85, 0.94, and 0.95, respectively. The second model focused on recognizing the absence of non-significant stenosis and significant stenosis showing per segment, vessel, and patient accuracy of 0.80, 0.76, and 0.75, respectively. Lee et al. developed an AI model that showed diagnostic accuracies of 92.3% and 88.4% at the patient and vessel levels. The model also had a superior agreement in binary stenosis classification at both patient and vessel levels with Cohen’s kappa coefficient of 0.79 vs. 0.39 and 0.70 vs. 0.40 (p<0.0001) [59].

In addition to detecting stenosis, various studies have demonstrated other uses of AI. One of them is the evaluation of Coronary Artery Calcium Score using AI. Wolternik and Ink demonstrated an automated algorithm's impact in determining CAC in a cohort of 250 patients who underwent CCTA. The authors constructed a CNN algorithm that generated a sensitivity of 0.72 for the evaluation of CAC. They also observed an interclass correlation of 0.94 between the CAC identified from CCTA and the standard evaluation of CAC. Identifying the plaque composition on imaging can impact patient management [60]. Calcified plaques have a better prognosis compared to fibrous plaques. AI models can help detect the plaque phenotype in a short period. An integrated approach using radiomics and ML has been developed to characterize plaques.

Using radiomics, various parameters can be derived from standard images that make a comprehensive assessment of the plaque. Zreik et al. have developed an algorithm for discerning plaque morphology by using 3D model CNN. Evaluation of 65 patients out of 95 revealed an accuracy of 0.85 in differentiating whether a plaque is present or not and the accuracy between plaque types was shown to be 0.77. ML uses algorithms to combine big data for ultimate high prediction in the computer science field. Prior research has indicated that ML can improve the prediction value of conventional scores for myocardial ischemia and mortality. Some techniques such as intravascular ultrasound (IVUS), optical coherence tomography, and near-infrared spectroscopy (NIRS) are used as histopathologic features of coronary atherosclerosis.

In one analysis, the specialized use of IVUS and NIRS in plaque characteristics identification was assessed, such as lipid-rich necrotic core [61]. However, the reports of the results intermingled with both underestimations, which were reported to be falsely enlarged in specific scenarios or overestimated, in calcifications of atherosclerosis shown in CCTA. The first line of CCTA was integrated in the United Kingdom in 2016 by the National Institution for Health and Care Excellence. It was later incorporated by the European Society of Cardiology in 2019. CCTA was selected to be the first-line tool in the assessment and evaluation of chronic coronary syndrome as well as chest pain [61]. It was demonstrated in the Evaluation of Integrated Cardiac Imaging in Ischemic Heart Disease study that the other types used as tools for CAD patients were positive emission tomography, single photon emission CT, echocardiography, and cardiac magnetic resonance.

The Prospective Multicenter Imaging Study for Evaluation of Chest Pain and the Scottish Computed Tomography of the HEART Trial have analyzed the utility of CCTA in predicting CAD, and it was observed that it may tip off the scale more than the traditional blood tests and medical standard care. As the SCOT-HEART followed up with CCTA in clinical setup for five years, it was reported that the incidence of myocardial infarction, and death by CAD were significantly reduced. However, it was reported by PROMISE trials that it did not play a role in improving clinical outcomes without using a functional test. They concluded that, based on the meta-analysis of CCTA used in patients with stable chest pain in comparison to traditional care, there was a 31% reported reduction in myocardial infarction and an absolute reduction in myocardial infarction rates of 1.8 events per 1000 patient-years. In addition to improving diagnostic efficacy, it is deemed a better strategy for placement of CCTA preceding the diagnostic invasive testing for suspected CAD, which has shown a significant reduction in diagnostic costs and angiography. With the need for emergency care ruled out, the use of CCTA on low-intermediate risk patients presenting with acute chest pain has been proven to be both safe and rapid, consuming less time and producing faster results, thereby enabling patients to be diagnosed early on, which considerably reduced hospital length of stay.

Another form of CAD-related prediction is coronary artery calcium scoring. This tool is used as a predictor of future coronary events by classifying the patient as a high-risk or low-risk category. Moreover, it can identify the plaque morphology and its composition, including non-calcified and calcified plaque, and can detect the severity of coronary stenosis. This tool can be used as a follow-up course of the condition with the treatment [62].

Clinical uses of radiomics, AI, and ML in CCTA

Coronary Artery Disease Revascularization

It is anticipated that CCTA will become the method of choice for excluding stable CAD [63]. A complete automation of the imaging process is urgently required to accommodate the dramatically increased workload. Among the many possible applications of AI is data collection, processing, and interpretation, which includes data acquisition via activities such as patient scheduling and preparation [63].

Quantification of Coronary Calcium

The detection and measurement of coronary calcium have a troubled history from a medical perspective. There have been some recent doubts about the relevance of assessing coronary calcium levels, which were formerly considered an essential component for detecting and forecasting CAD. In recent years, it has witnessed a reemergence. Still, coronary calcification's possible use as a gatekeeper is a matter of continuing controversy. Contrast is indeed necessary to make lipid-rich plaques visible. However, studies show that a high Agatston score (400 or above) is associated with an increased risk of cardiovascular disease and mortality. The usual method for assessing coronary calcium involves imaging with a single breath-hold using high-pitch EKG-gated scanning. It is well acknowledged that specialist scanning methods may automatically detect calcified plaques [64], but obtaining a trustworthy Agatston score for risk assessment often requires human intervention. Having AI models that can predict the Agatston score on native chest CT scans without EKG-gating or fixed tube voltage would be beneficial since these scans are rather common. With or without EKG gating, a new algorithm can accurately forecast coronary artery calcium scores in different kinds of CT images. This approach has proven to be quite accurate across various test formats by eliminating the need for a specific calcium-scoring exam.

In addition, new dual-energy and photon-counting detector CT scanners suggest using virtual non-contrast-enhanced pictures from a conventional CCTA to identify calcification. This method avoids excessive radiation exposure [65]. Recently, CCTA reconstructions of PureCalcium outperformed the CAC score in virtual non-contrast (VNC) reconstructions, according to one study. It is best to build robust models for fully automated CAC scoring in this setting.

CT-Fractional Flow Reserve (CT-FFR)

From a therapeutic perspective, CT-FFR is very beneficial since it provides information on hemodynamics, namely the importance of coronary stenoses, that extends beyond morphology alone. Despite the positive early reviews and publications in clinical research, more and more people are beginning to question CT-FFR and FFR measurement's practical use [66]. The results of studies are becoming increasingly conflicting as they approach clinical practice, and it seems that extra hemodynamic analysis is not having much of an effect.

The automated determination of CT-FFR is now possible with the help of AI algorithms. But there has been a holdup and much additional money has been spent since only one supplier has obtained this accreditation. Due to patent constraints, many on-site machine learning methods are only legal for use in academic settings. Despite this, these techniques are fast and long-lasting, enabling us to evaluate the 3D dynamics of coronary artery blood flow quickly and thoroughly [67]. Coronary segments that have undergone alterations in blood flow are color-coded for easy identification in this method, which allows for a rapid three-dimensional evaluation of the coronary architecture. Yet, neither the automatic transmission to CCTA nor the integration of these data into the therapeutic process has been accomplished.

CT Profusion

Imaging and assessing myocardial CT perfusion has therapeutic potential as it may help detect coronary stenoses that significantly affect blood flow and are amenable to invasive treatment. However, there is still a downside to the present dynamic CT perfusion protocols: they use a lot of radiation. Because of this, improving techniques for analyzing myocardial perfusion data from standard coronary CT angiography datasets is crucial. Another potential strategy is to use AI algorithms to transform low-dose dynamic perfusion data into comprehensible visuals.

A recent study sought to identify an alternative to dynamic imaging. Through DL, they have deduced the patterns of myocardial enhancement that, when detected by invasive fractional flow reserve, signify the existence of hemodynamically substantial stenosis in the epicardial coronary arteries [68]. Using DL analysis, improved diagnostic performance in identifying individuals with functionally significant coronary artery stenosis was achieved by measuring the degree of luminal narrowing in intermediate-degree coronary stenosis. Compared to classification based on DS alone (AUC=0.68), the proposed method improved differentiation (AUC=0.76). By using DL for CT image reconstruction, low-dose CT dynamic myocardial perfusion has been significantly improved.

Recent research has presented a DL method that, when compared to conventional hybrid iterative reconstruction techniques, may successfully reduce image noise by around 20%. Additional reductions in radiation dose may result from this noise reduction [69]. Incorrect temporal frame alignment is a major source of worry in CT dynamic perfusion imaging, leading to incorrect CT myocardial blood flow estimations. To address this issue, a study has suggested using DL for picture registration. After limiting local tissue movements in the left ventricle, the researchers found that their recommended technique efficiently captured dynamic cardiac perfusion sequences. The pictures' quality, namely the absolute CT values throughout the series, was maintained. Furthermore, compared to conventional image registration methods, the DL-based technique dramatically reduced processing time, requiring just a few seconds. CT-FFR and coronary calcium measurement, along with CT myocardial perfusion imaging, are not yet part of the routine clinical reporting process. Nevertheless, AI algorithms are expected to play a role in facilitating this integration soon [69].

Scar Imaging

One of the most important uses of contemporary cross-sectional cardiovascular imaging is the noninvasive identification of the existence and extent of myocardial scars. Undoubtedly, CCTA has fallen far behind other methods, including nuclear imaging techniques and cardiac MRI, mostly because of the much lesser distinction between scar tissue, viable tissue, and normal myocardium. Nevertheless, ML has a substantial opportunity to narrow this disparity. One study has reported favorable outcomes when using CNNs to identify subendocardial scarring from delayed-enhancement CCTA images [70]. The CNNs achieved a sensitivity of 91%, specificity of 88%, and accuracy of 89% compared to segmentations made by human experts. Their method included integrating CNN-based automatic left ventricle segmentation with topological data analysis to obtain geometric scar features.

Another study recently used a radiomics technique to completely automate the diagnosis of left ventricular scarring in delayed-enhancement CCTA. Out of the 93 radiomics characteristics that were computed, over two-thirds showed a significant correlation with the existence of myocardial scar. The pictures obtained at a voltage of 100 kV yielded the most effective ML classifier, namely a support vector machine with an AUC of 0.88. The core results in this research were determined by using CMR-based segmentations of left ventricular scar that were aligned with delayed-enhancement CT scans to measure scar areas accurately. This work offers more evidence that radiomics approaches can complement picture assessment conducted by human specialists. Additionally, it presents promising findings on the ability of ML to reduce subjectivity in image evaluation.

While more development and validation in other groups are necessary, these approaches demonstrate the feasibility of using delayed-enhancement CT imaging to accurately identify scarring in the left ventricular myocardium, providing proof of concept. In addition, initial radiomics methods have been used to identify myocardial scars in both non-contrast and contrast-enhanced CT imaging; however, more research is required to confirm their effectiveness [71,72].

Challenges and prospects

The integration of AI in the medical field shows a potential path for advancing the diagnosis and management of diseases, thereby improving patient outcomes through unconventional data processing systems and analysis tools. Nevertheless, several hindrances hamper its smooth amalgamation into clinical practice. Firstly, the ML models that are commonly known as “black boxes” do not have clinical applications. To simplify these models, more efforts are being made towards developing explainable ML models [73]. Also, limited external validation and sampling bias during model development can be challenging, especially when datasets lack representation and standardization. Data standardization is important for reliable analysis, particularly when varied sources such as multiple hospital systems need to be brought together. Additionally, concerns persist about unauthorized data access in the healthcare system. However, the possible integration of AI in healthcare systems offers a potential advancement for bettering clinical diagnosis and maximizing medical services provided that the challenges are addressed.

The intelligence framework constitutes a variety of techniques that allow machines to perform tasks that used to require human intelligence and effort. An important subfield of AI is ML, which includes algorithms that learn from experience and data or predictive decisions. In ML, DL is a dedicated task that uses various layers of neural networks to perform complex tasks. CNN, a type of DL algorithm, is well-suited for tasks involving image and pattern recognition. These are made with the alteration and analysis of graphic input in mind. CNNs are trained continually to identify and categorize the medical imaging data for prediction [74].

Challenges of AI-Based Image Analysis in CCTA

Re-evaluation of the financial models used by healthcare organizations is the primary step, and new methods for handling financial and monitoring matters, especially related to AI applications, are required. The use of AI-based models prompts for ethical inquiries [75]. The integration of AI and digital health technologies is beginning to be established by organizations like the National Institute for Healthcare Excellence [56]; these initiatives offer standards for assessing the efficacy of AI applications in healthcare environments. Furthermore, integrating AI requires continuous data collection within centralized databases to support algorithmic analyses. However, this would raise significant ethical considerations regarding data proprietorship, data use capacity, security, and algorithmic standards. Meanwhile, carefully considering data privacy and bias issues, including patient data, technology vendors, or healthcare organizations, is essential [75,56].

Future Perspectives on AI Integration in CCTA

AI has the potential to significantly improve precision medical pathways and change early CAD care with CCTA. AI can reduce the burden of CAD, facilitate myocardial ischemia care, and enhance picture quality. AI facilitates resuscitation dose reduction by aiding pathological and prognostic assessment and plays an important role in CCTA. Despite awareness of human interpreters, AI contributes to reducing fatigue and reporting time, especially as CCTA requirements increase to meet clinical guidelines [76]. Furthermore, the development of CCTA analysis to a precision medicine model requires detailed reports beyond coronary stenosis grading, including plaque characterization, measurement of hemodynamic effects, and conceivably advanced AI algorithms for automated CCTA image analysis after myocardial ischemia detection and integration with clinical criteria to generate patient-specific risk profiles. However, using AI in cardiac imaging requires clear regulation of medical and legal aspects and confidentiality concerns associated with data analysis [76]. Since they offer vital interpretability insights, broad collaborations across the medical and technical fields are essential to the future of AI-driven clinical innovation. Enhancing interpretability enhances knowledge and comprehension while advancing scientific research. To fully utilize AI, reliable data collection and access are essential [76].

## Conclusions

The integration of AI into CCTA is the next revolutionary step in the diagnosis and management of CAD. Through AI technologies like ML and DL, CCTA is significantly empowered to formulate better ways to analyze extensive imaging data more accurately and rapidly than before. AI helps improve diagnosis, provide personalized treatment plans, and potentially enhance patient outcomes by complementing the analysis of coronary pathology, which is impossible in the context of traditional methods. Despite the huge benefits of introducing AI in CCTA, some challenges hinder the practical application of AI. These challenges involve data privacy, transparency of AI algorithms, and standardization across different platforms and institutions. It is imperative to overcome these challenges to establish trust in AI-assisted diagnostics systems and capitalize on their full capacity. Going forward, continual efforts on the part of clinicians, developers, and regulators are required to get the most out of AI-assisted imaging. This involves training clinicians about working with AI, rendering AI more transparent to the final user, and developing abidance to guide the ethical use of AI. Ultimately, AI and medical imaging will be integrated seamlessly into how CAD patients are cared for, immensely improving clinical outcomes and enhancing workflow efficiency.
